# Prevalence and associated factors of probable depression amongst pregnant and parenting young females: a comparison of adolescents and young adults in rural South Africa

**DOI:** 10.3389/frcha.2023.1200759

**Published:** 2023-10-16

**Authors:** K. P. Seakamela, R. G. Mashaba, C. B. Ntimana, M. O. Mbombi, J. Tlouyamma, P. Mphekgwana, R. Nemuramba, K. Mothapo, L. Muthelo, L. N. Mabila, I. Dhau, E. Maimela

**Affiliations:** ^1^ DIMAMO Population Health Research Centre, University of Limpopo, Polokwane, South Africa; ^2^ Department of Nursing Science, University of Limpopo, Polokwane, South Africa; ^3^ Department of Computer Science, University of Limpopo, Polokwane, South Africa; ^4^ Research Administration and Development, University of Limpopo, Polokwane, South Africa; ^5^ Department of Pharmacy, University of Limpopo, Polokwane, South Africa; ^6^ Department of Geography and Environmental Studies, University of Limpopo, Polokwane, South Africa; ^7^ Department of Public Health, University of Limpopo, Polokwane, South Africa

**Keywords:** financial support, socio-demographic, HIV status, alcohol consumption, depression

## Abstract

**Background:**

Pregnant teenagers have been reported to have an increased likelihood of experiencing depression than their non-pregnant peers. There is little research on the risk factors for depression in rural Black adolescents and young adults, especially after the Covid-19 pandemic. Therefore, the current study aimed to identify the prevalence of probable depression and associated factors amongst pregnant and parenting young females.

**Method:**

The study was a cross-sectional design, consisting of 362 pregnant and parenting adolescents and young adults aged 14–22. The study used the Edinburgh Postnatal Depression Scale (EPDS) to measure probable depression. Data were analyzed using Statistical Package for Social Sciences SPSS, version 27.0.

**Results:**

The study found that the overall prevalence of probable depression was 42.8%. The study also found a relationship between alcohol consumption, lack of financial support, unplanned pregnancy, and probable depression in pregnant and parenting adolescents. The prevalence of unplanned pregnancy in the present study was 81.8%.

**Conclusion:**

Furthermore, the present study indicated that participants from low socio-economic status families and those who were HIV positive were at a greater risk of depression in both groups. Therefore, we recommend that measures be put in place for early detection and treatment of depression and that social support be given to adolescent mothers.

## 1. Introduction

Depression is a leading mental health condition that presents symptoms such as loss of interest, guilt or low self-worth, disturbed sleep, irregular eating, fatigue, and lack of focus (1, 2). It is at the forefront of public health concerns affecting approximately 280 million people worldwide (3). Pregnant women in low- and middle-income countries (LMICs) are reportedly more likely to experience depression than those in developed countries (4, 5). Pregnant women have a significantly higher rate of depression, according to prior studies, with between 11% and 18% prevalence globally (6, 7). According to the World Health Organization, 1 million girls under the age of 15 and 16 years give birth each year, with the majority of these women living in LMICs (8, 9). In addition to the developmental dangers of adolescence, the load of pregnancy or parenting during this time increases the likelihood of mental health issues including depression (10–12). Teenage pregnancy and mental health issues represent a “double vulnerability” for adverse consequences in both teens and older women (13). Several studies also narrated that in Sub-Saharan Africa (SSA), young females’ pregnancy rates are among the highest, with associated poor health outcomes, such as maternal mortalities, injuries, and adverse newborn outcomes (14, 15). The literature revealed that during the COVID-19 pandemic, between April 2020 and March 2021, South Africa reported that more than 23,000 girls under 18 gave birth, with 934 of those births occurring to children under 14 years (16). Hence the COVID-19 pandemic was reported to have caused a spike in depression and anxiety among pregnant mothers (17, 18).

Several studies conducted in South Africa showed a high prevalence of depression among parenting adolescents (Khayelitsha 39%, Witzenburg 50.3%, and KwaZulu-Natal 44.9%) (12, 19, 20). The contributing factors that result in the higher percentiles of depression in adolescents are reported to be poverty, abuse, victimization, and lower education levels (4). In South Africa, recent Department of Basic Education statistics revealed that 33% of these teen mothers do not return to school after pregnancy (20). Thus, lack of education diminishes opportunities for advancement available to teen mothers, perpetuating inequality and poverty that made them vulnerable in the first place (20). Statistics South Africa (Stats SA) has further shown that high rates of teenage pregnancies are seen in the more rural provinces like the Eastern Cape, Mpumalanga, and Limpopo, establishing the direct link between pregnancies and poverty (21). Hence the current study was conducted in the rural communities of Limpopo province which are predominated by poverty, unemployment, and poor education. More importantly, most households depend on social grants (22). Therefore, there is a need to identify the prevalence of depression and associated factors among pregnant mothers and young females in these communities. This will assist in the development of strategies to assist these vulnerable groups to overcome mental health issues.

Studies have been conducted on associated risk factors of depression in pregnant and postpartum adolescents (23–25). Dysfunctional family structures, low socioeconomic status, a lack of family support, social isolation, a history of physical and sexual abuse, and partner neglect are all risk factors for depression in pregnant and postpartum teenagers (23, 26–28). Furthermore, motherhood in young females is associated with less support, stigma, discrimination, gender inequality, and derailed educational aspirations, which increase the risk of depression (29). The impact of postpartum depression on parenting young females includes regrets of mothering as they see it as punishment, tend to feed less and use emotional coping styles such as substance abuse and suicide attempts (4, 29).

Depression amongst pregnant and postpartum adolescents has been widely researched. However, there is little literature on the prevalence and associated risk factors of depression between adolescents and young adults. Therefore, the current study aimed to investigate the prevalence and associated factors of depression amongst pregnant adolescents and young adults in Limpopo province, South Africa.

## 2. Methodology

### 2.1. Study setting

The study was conducted at the DIMAMO Health and Demographic Surveillance Site (HDSS) in the Capricorn District of the Limpopo Province of South Africa. With 57 settlements and over 100,000 residents, the DIMAMO areas are rural and semi-urban parts of the HDSS. Speaking Northern Sotho is the dominant language in this region. Black people make up a sizable section of the population and are of low socioeconomic status and low educational level. The study area includes 11 PHCs and one tertiary hospital that provides health care services to the residents within the surveillance region. Data was collected from pregnant and parenting adolescents in the PHCs around the DIMAMO HDSS.

### 2.2. Population and sampling

About eleven (11) clinics that provide services to residents in the DIMAMO HDSS were used to recruit participants for this cross-sectional study. The sample size was calculated using the Cochrane sample size equation and the minimum sample size for the study was 152 and 362 participants were recruited (30). Of the 362 participants who were recruited, 121 were pregnant, and 232 were parenting adolescent females aged between 14 and 22 years were included. The study also included 7 participants who had abortions and fit the age criteria. However, because the study's focus is on present parenting and pregnant females, these participants were omitted from the final analysis.

### 2.3. Data collection

The instrument used to gather data was a structured questionnaire constructed based on the literature study objectives and research questions (31). The questionnaire included, amongst others, questions on socio-economic, demographics, mental health determinants, pregnancy and parental care, and substance use. Alcohol consumption and smoking were categorized by current use status (past 30 days). The questionnaire was written in English and translated into the local language to enhance the quality of responses. Trained field workers administered the questionnaire in a private area to maintain the confidentiality and anonymity of the participants. All field workers signed a non-disclosure agreement and were trained on research ethics and consent. The questionnaire had ten self-reported questions linked to clinical cognitive symptoms of depression. The assessment of probable depression among women was performed by assigning the scores to responses from the ten self-reported questions (refer to Supplementary Appendix 1). The questionnaire was drafted and sent to the research team with a validation form for validation processes. The form had scores measuring each question's relevance [1 = not relevant, 2 = somewhat relevant, 3 = quite relevant, 4 = highly relevant] and clarity [1 = not clear, 2 = somewhat clear, 3 = quite clear, 4 = highly clear]. These scores were used to calculate the level of agreement and subsequently content validity index and a mean content validity index of above 0.80 was considered acceptable (32). In addition, the questionnaire was further validated by conducting a pilot survey among the first 6 participants from three different clinics. The questionnaire was considered valid after it was able to measure the intended parameters when repeated several times.

To correctly set the depression risk score, the study adopted the Edinburgh postnatal depression scale (EPDS). The EPDS tool is used to assign, assess, and identify women who may experience probable depression during or after pregnancy. According to the literature, the sensitivity, specificity, and positive predictive value of EPDS are increased to 86%, 78%, and 73%, respectively, with a cut-off score of 13 or higher (1, 2). As shown in Table 1, each question has four responses with an assigned score of 0–3, having a combined score falling between 0 and 30 (3). Answers for questions 1, 2, and 4 are scored in ascending order of 0–3, while questions 3, 5–10 have the scores assigned in descending order of 3–0. Probable depression (EPDS ≥10) was the result of the sum of the scores from the questions, with categories viz. not depressed (EPDS = 0–9), mild (EPDS = 10–13), moderate (EPDS = 14–20), severe (EPDS = 21–27) and extremely severe (EPDS = 28+) (33, 34).

**Table 1:** Sociodemographic information amongst postnatal and prenatal adolescent and young adults.

Variables	Categories	Adolescents (*n* = 253)		Young adults (*n* = 102)	
		Postnatal	Prenatal	Postnatal	Prenatal
		*N* (%)	*N* (%)	*N* (%)	*N* (%)
Marital status	Unmarried	152 (62.6%)	91 (37.4%)	76 (77.6%)	22 (22.4%)
	Married	4 (40.0%)	6 (60.0%)	1 (25.0%)	3 (75.0%)
Highest level of education	None	2 (50.0%)	2 (50.0%)	1 (50.0%)	1 (50.0%)
	Primary	3 (50.0%)	3 (50.0%)	0 (0%)	0 (0%)
	Secondary	141 (61.8%)	87 (38.2%)	61 (80.3%)	15 (19.7%)
	Tertiary	10 (66.7%)	5 (33.3%)	15 (62.5%)	9 (37.5%)
Employment status	Employed	1 (50.0%)	1 (50.0%)	3 (100.0%)	0 (0.0%)
	Unemployed	154 (61.6%)	96 (38.4%)	74 (74.7%)	25 (25.3%)
Alcohol consumption	Yes	17 (51.5%)	16 (48.5%)	17 (73.9%)	6 (26.1%)
	No	139 (63.2%)	81 (36.8%)	60 (75.9%)	19 (24.1%)
Smoking	Yes	1 (50.0%)	1 (50.0%)	2 (66.7%)	1 (33.3%)
	No	154 (61.6%)	96 (38.4%)	75 (75.8%)	24 (24.2%)
Financial support by partner	Yes	95 (56.9%)	72 (43.1%)	55 (72.4%)	21 (27.6%)
	No	60 (70.6%)	25 (29.4%)	22 (84.6%)	4 (15.4%)
Partner violence	Yes	15 (60.0%)	10 (40.0%)	5 (83.3%)	1 (16.7%)
	No	139 (61.5%)	87 (38.5%)	72 (75.0%)	24 (25.0%)
Planned pregnancy	Yes	27 (65.9%)	14 (34.1%)	20 (80.0%)	5 (20.0%)
	No	129 (60.8%)	83 (39.2%)	57 (74.0%)	20 (26.0%)
HIV Status	Positive	7 (53.8%)	6 (46.2%)	3(100.0%)	0(0.0%)
	Negative	146(62.9%)	86(37.1%)	73(76.0%)	23(24.0%)

### 2.4. Data analysis

Data were analyzed using Statistical Package for Social Sciences (SPSS), version 27.0 (1). The socio-demographic data underwent a descriptive analysis. The participants ranged in age from 14 to 22 years. Continuous data were given as means and standard deviations, whereas categorical data were presented as frequencies and percentages (%). Chi-square was conducted to examine the relationship between probable depression and sociodemographic factors (35).

### 2.5. Ethical considerations

The University of Limpopo Turfloop Research Ethics Committee (TREC) granted ethical approval for the project. Permission to conduct the study was also granted by the tribal authority and the Department of Health in Limpopo Province. The parent's consent was solicited in cases where the participant was below the consenting age of 18.

## 3. Results

Table 1. Most of the postnatal adolescents and young adults were not married. Within the education levels, most participants had formal education, with the number observed in the secondary school level for postnatal adolescents and young adults being (61.8% and 80.3% respectively), while prenatal adolescents and young adults had (38.2% and 19.7% respectively). Young adults in the postnatal category had the highest percentage of unemployment compared to adolescents in both postnatal and prenatal. Half of the postnatal and prenatal adolescents were alcohol consumers, whereas 74% of postnatal young adults consumed alcohol. Most of our participants reported that they had financial support while only a quarter reported not receiving it. Most adolescents, compared to young adults, reported having experienced domestic violence. More than three-quarters (80%) of our participants reported their pregnancy as unplanned, with the highest percentage observed in postnatal adolescents. Most of our participants were HIV negative, with only <5%, primarily adolescents reported to be living with HIV.

**Figure 1:**
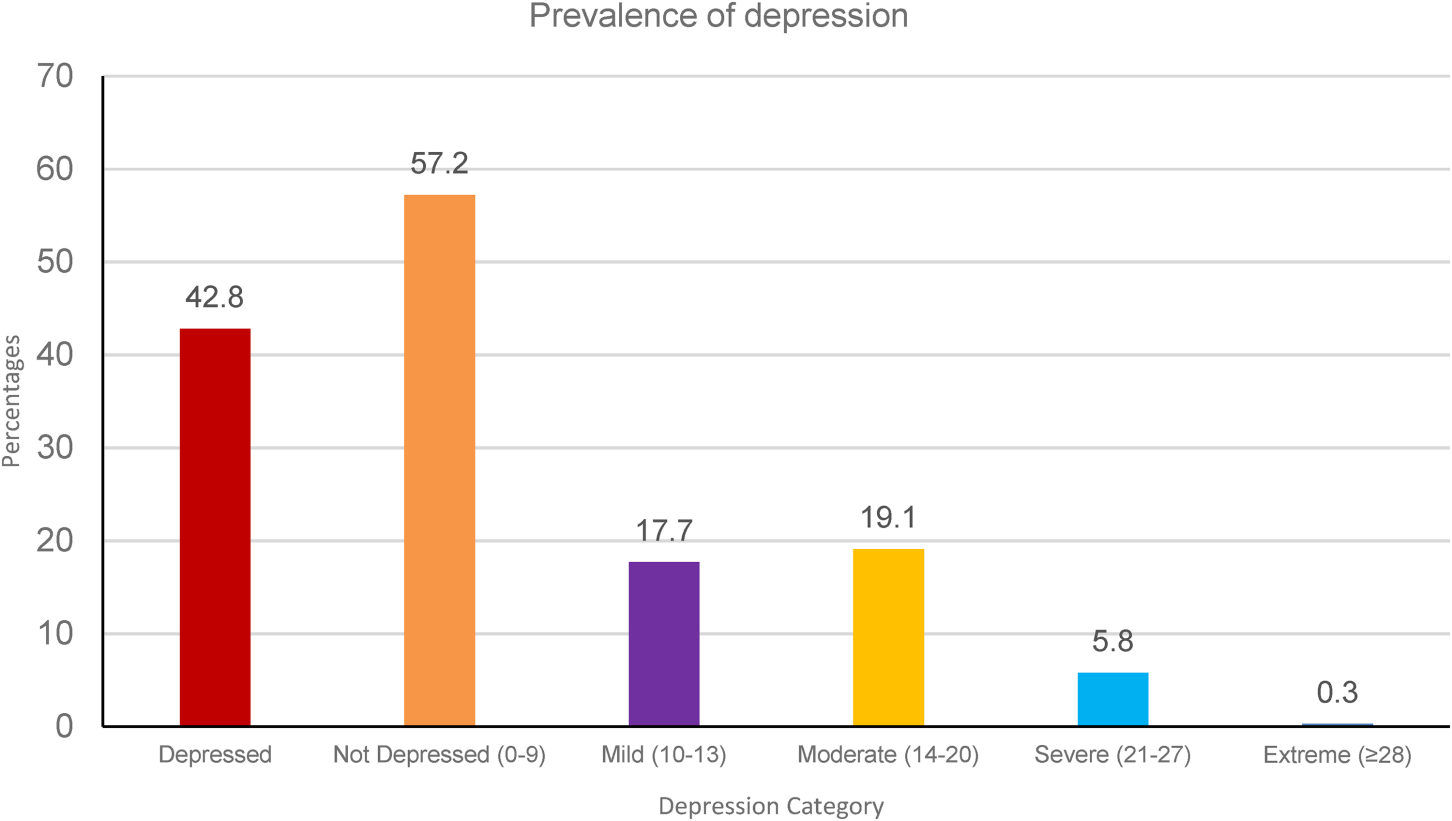
Depression severity, the figure in the brackets denotes depression severity bands.

The EPDS scale was used to assess the prevalence of probable depression in our study population, and we found 42.8% to be probably depressed. When assessing the severity of depression, we found 17.7%, 19.1%, 5.8%, and 0.3% to be mildly, moderately, severely, and extremely depressed, respectively (see Figure 1).

Table 2. There was a significant difference in alcohol consumption criteria among adolescents, with most (63.6%) of the participants who were alcohol consumers being at a greater risk of depression (*p = 0.016*). Financial support was associated with probable depression for both adolescents and young adults, where most of the participants who did not have financial support (61.2% and 57.7% respectively) were at a greater risk of depression (*p < 0.001*). There was a significant difference in planned pregnancy and HIV status, 48.6% of adolescents who did not plan their pregnancy were depressed, and those who were living with HIV were at a greater risk of depression (76.9%) (*p = 002* and *p = 0.014* respectively).

**Table 2:** Association between the risk factors and depression amongst adolescents and young adults.

Variables	Categories	Adolescents (n=253)			Young adults (n=102)		
		Not depressed	Depressed	*p*-value	Not depressed	Depressed	*p*-value
		*N* (%)	*N* (%)				
Marital status	Unmarried	134 (55.1)	109 (44.9)	0.354	60 (61.2)	38 (38.8)	0.578
	Married	7 (70.0%)	3 (30.0%)		3 (75.0%)	1 (25.0%)	
Highest level of education	None	3 (75.0%)	1 (25.0%)	0.586	0 (0.0%)	2 (100.0%)	0.166
	Primary	2 (33.3%)	4 (66.7%)		0 (0%)	0 (0%)	
	Secondary	127 (55.7%)	101 (44.3%)		49 (64.5%)	27 (35.5%)	
	Tertiary	9 (60.0%)	6 (40.0%)		14 (58.3%)	10 (41.7%)	
Employment status	Employed	2 (100.0%)	0 (0.0%)	0.204	2 (66.7%)	1 (33.3%)	0.859
	Unemployed	138 (55.2%)	112 (44.8%)		61 (61.6%)	38 (38.4%)	
Alcohol consumption	Yes	12 (36.4%)	21 (63.6%)	0.016	14 (60.9%)	9 (39.1%)	0.920
	No	129 (58.6%)	91 (41.4%)		49 (62.0%)	30 (38.0%)	
Smoking	Yes	1 (50.0%)	1 (50.0%)	0.865	3 (100.0%)	0 (0.0%)	0.167
	No	140 (56.0%)	110 (44.0%)		60 (60.6%)	39 (39.4%)	
Financial Support by Partner	Yes	107 (64.1%)	60 (35.9%)	<0.001	52 (68.4%)	24 (31.6%)	<0.001
	No	33 (38.8%)	52 (61.2%)		11 (42.3%)	15 (57.7%)	
Partner Violence	Yes	11 (44.0%)	14 (56.0%)	0.211	3 (50.0%)	3 (50.0%)	0.541
	No	129 (57.1%)	97 (42.9%)		60 (62.5%)	36 (37.5%)	
Planned pregnancy	Yes	32 (78.0%)	99 (22.0%)	0.002	19 (76.0%)	6 (24.0%)	0.092
	No	109 (51.4%)	103 (48.6%)		44 (57.1%)	33 (42.9%)	
HIV Status	Positive	3 (23.1%)	10 (76.9%)	0.014	0 (0.0%)	3(100.0%)	0.058
	Negative	134(57.8%)	98(42.2%)		60(62.5%)	36(37.5%)	

Table 3. Parents/guardians’ reaction to pregnancy was associated with probable depression, with most of the adolescents who reported their parents/guardians to be disappointed by their pregnancy being at a greater risk of depression (63.5%) (*p < 0.001*). The same association distribution was observed in adolescents who reported teenage pregnancy to be viewed as a shameful incident in their communities (55.1%) (*p* = 0.011). For both adolescents and young adults, not having food for a day was associated with probable depression (*p < 0.001* and *p = 0.007* respectively). The same significant difference was observed in both adolescents and young adults who reported a shortage of food for their children (*p < 0.001* and *p = 0.031* respectively).

**Table 3:** The association between depression and food security amongst adolescents and young adults.

Variables	Categories	Adolescents (*n* = 253)			Young adults (*n* = 253)		
		Not depressed	Depressed	*p*-value	Not depressed	Depressed	*p*-value
		*N* (%)	*N* (%)		*N* (%)	*N* (%)	
What were your parents/guardians’ reactions’ when they found out you were pregnant?	Very disappointed	31 (36.5%)	54 (63.5%)	<0.001	12 (48.0%)	13 (52.0%)	0.270
	Somehow disappointed	58 (59.2%)	40 (40.8%)		23 (63.9%)	13 (36.1%)	
	Happy	52 (74.3%)	18 (25.7%)		27 (67.5%)	13 (32.5%)	
Based on your understanding, what is your view on teenage pregnancy in your area	Very shameful	48 (44.9%)	59 (55.1%)	0.011	32 57.1%	24 42.9%	0.329
	Shameful	33 (66.0%)	17 (34.0%)		7 (53.8%)	6 (46.2%)	
	Acceptable	60 (62.5%)	36 (37.5%)		23 (71.9%)	9 (28.1%)	
I was in control of the decisions being made about my prenatal care	Yes	100 (56.2%)	78 (43.8%)	0.386	47 (60.3%)	31 (39.7%)	0.852
	No	28 (60.9%)	18 (39.1%)		10 (66.7%)	5 (33.3%)	
	Not sure	13 (44.8%)	16 (55.2%)		6 (66.7%)	3 (33.3%)	
My values and beliefs were respected by prenatal care providers)	Yes	108 (58.7%)	76 (41.3%)	0.223	46 (58.2%)	33 (41.8%)	0.282
	No	13 (54.2%)	11 (45.8%)		5 (62.5%)	3 (37.5%)	
	Not sure	20 (44.4%)	25 (55.6%)		12 (80.0%)	3 (20.0%)	
In the last 12 months, was there a time you or other members of your household went without eating for a whole day because of a lack of money or other resources	Yes	13 (24.1%)	41 (75.9%)	<0.001	1 (14.3%)	6 (85.7%)	0.007
	No	126 (64.3%)	70 (35.7%)		62 (65.3%)	33 (34.7%)	
	Don’t know	2 (66.7%)	1 (33.3%)		0 (0%)	0 (0%)	
In the last 12 months did you ever cut the size of the children’s meals or did the children skip a meal	Yes	17 (29.3%)	41 (70.7%)	<0.001	4 (33.3%)	8 (66.7%)	0.031
	No	124 (63.9%)	70 (36.1%)		59 (65.6%)	31(34.4%)	
	Don’t Know	0(0.0%)	1(100.0%)		0(0%)	0(0%)	

## 4. Discussion

The purpose of the study was to find out the prevalence of probable depression and its contributing factors amongst pregnant and parenting adolescents and young adults. The prevalence of probable depression in the total population was 42.8%. When comparing the prevalence of probable depression between prenatal adolescents and postnatal adolescents, a significantly higher prevalence of probable depression was noted in postnatal adolescents. In agreement with the present study, Oladedji et al., (2019), reported depression to be more prevalent in younger (16 years and below) pregnant and parenting adolescents and decreased with an increase in age (36). This may suggest that as pregnant and parenting adolescents grow older, they are more likely to be able to deal with the pressures of life. The present study found a relationship between alcohol consumption and probable depression in pregnant and parenting adolescents. Pregnant teenagers were reported to go through an identity crisis, suffer from low self-esteem, and feel lost for the future (37). Guilt brought on by rejection from family, friends, partners, and society makes them feel less interested in participating in discussions and self-isolating. As a result, they experience various pressures, leading to probable depression (37). In addition, post-natal probable depression was reported to be common in teenagers because of lack of support, isolation from peers and/or families, and financial pressures and social attitudes (38). In agreement with the present study, Tele et al., (2022) reported similar findings (29). Substance use including alcohol consumption was reported to be a risk factor associated with a higher depression score (39).

In the present study alcohol consumption was associated with probable depression in adolescents. In accordance with the present study Edwars et al., (2014), reported alcohol consumption to be associated with an increase in depression symptoms (40). The positive association between alcohol consumption and probable depression in adolescents might be that they tend to drink alcohol as a coping mechanism (41)]. However, there was no association between alcohol consumption and probable depression in young adults. Public health education interventions that will take into account developmental needs for support, behaviour during pre and postpartum, and the influence of partners and friends on substance use are needed (42).

In the current study, probable depression was associated with the partner's lack of financial support. The lack of spousal financial support for the pregnant and or parenting adolescent may lead to probable depression. In a study conducted by Thompson and Ajayi, (2016), depression was associated with a lack of financial, psychological, and preparedness to deal with the demands of pregnancy (39). The current study also found that the view on pregnancy in the community and how the parents reacted to the pregnancy was also associated with probable depression. However, this association was found only in adolescents and not in young adults. Krugu et al., (2017) reported that parents and or guardians are not always happy with teenage pregnancy, Since teenagers value their opinions, it may lead to depression which is evident in our current study (43). Other studies have highlighted the concerns of parents about their teenage daughters’ health and educational achievements, which Rachakonda et al., (2014), and Nkosi and Pretorius, (2019) reported a negative relationship (44, 45).

Both the pregnant and parenting adolescents and young mothers report that they and members of their household went without eating for a whole day because of a lack of money or other resources in the past 12 months, and this was positively associated with probable depression. In addition, they reported having cut, at least once, their children's meals or skipped meals due to finances; this was positively associated with probable depression. These may partially speak to the socioeconomic status of the household. The area from which the study was conducted is of rural and semi-rural settings and low socioeconomic status (46). Therefore, the lack of food may be reflective of the socioeconomic status of the area. Although the two questions asked in this study do not give the complete picture of the socioeconomic status of the participants, other studies have reported an association between low socio-economic status and depression in adolescents and young adults (47–49).

In the present study, most adolescents who had unplanned pregnancies had a higher risk of probable depression. The same was not the case with young adults. The prevalence of unplanned pregnancy in the present study was 81.8%. The increased prevalence of unplanned pregnancy in adolescents may be a result of a lack of maturity to make positive decisions about their lives, gender power dynamics that prevent them from negotiating safe sex, older partners who out-negotiate them, and the likelihood of sexual abuse which they may be prone to due to age. This may not be the case in young adults as they assume responsibility and maturity to make good decisions and avoid abuse hence in this study, no association between probable depression and unplanned pregnancy was found in young adults. Several studies have reported unplanned pregnancy to be associated with depression (19, 27, 39, 50).

Furthermore, the present study indicated that both adolescents and young adults who were HIV-positive were more likely to be depressed. A study by Thompson and Ajayi, (2016) reported that pregnancy with a medical condition such as HIV, hypertension, and diabetes mellitus was a risk factor for depression (39). Similarly to this, additional research revealed a connection between HIV and depression (19, 51). These illnesses alter people's lives and limit their mobility and independence thus causing depression which results in suicide attempts (37). Suicide is the tenth most frequent cause of death in this age range in Africa and is associated with depression and anxiety (52, 53). Depression manifests in the form of mood, disruptive behavioural, substance abuse, and psychosomatic disorders (52). In adolescents and young adults living with HIV, depression is triggered by social stigma, underlying disability, physical or verbal abuse, and denial of their HIV status or non-disclosure (54).

## 4. Limitations

The results of the current study should be interpreted with caution due to the following limitations. Firstly, the current study was cross-sectional in design which limits the quantification of risk factors of depression among pregnant and parenting groups (adolescents and young adults). Secondly, the EPDS scale cannot be used as a diagnostic tool for depression. Thirdly, the depression symptoms data was self-reported which could have caused an underestimation or overestimation of the prevalence of probable depression.

## 5. Conclusion

This is the first study to investigate the prevalence of probable depression and its associated risk factors in the prenatal and postpartum compared to adolescents and young adults in the DIMAMO HDSS. The study found social issues associated with probable depression among black adolescents and young adults residing in rural regions of the South African province of Limpopo. Overall, 42.8% of our participants were depressed. Probable depression was associated with sociodemographic factors including alcohol consumption and unplanned pregnancy in the adolescent group and financial support for adolescents and young adults. Probable depression was also associated with parents’ reaction to pregnancy, and the community view on pregnancy for adolescents whereas shortage of food for participants and their children is associated with probable depression for both adolescents and young adults. HIV status was associated with probable depression in both adolescents and young adults. Factors associated with probable depression were more prevalent among postnatal as compared to prenatal adolescents and young adults. We, therefore, recommend that more support be given to adolescent mothers as they may be overwhelmed by the responsibility of taking care of a newborn. Reaching out to loved ones for social support should be encouraged since early diagnosis and treatment of depression and associated medical disorders are crucial.

## Data Availability

The raw data supporting the conclusions of this article will be made available by the authors, without undue reservation.
