# Supplementary material for: Prevalence and associated factors of probable depression amongst pregnant and parenting young females: a comparison of adolescents and young adults in rural South Africa
Source: Front Child Adolesc Psychiatry. 2023 Oct 16;2:1200759. doi: 10.3389/frcha.2023.1200759 (PMC11748796; doi:10.3389/frcha.2023.1200759)
Supplement: Supplementary file 1 [file Table1.docx]

**Prevalence** **and Associated factors of probable depression amongst pregnant and parenting young females: a comparison of adolescents and young adults in rural South Africa**

Seakamela K.P*^1^, Mashaba R.G^1^, Ntimana C.B*^1^, Mbombi M.O^2^, Tlouyamma J ^1,3^, Mphekgwana P ^4^, Nemuramba R^1^, Mothapo k^1^, Muthelo L^2^, Mabila L.N^5^, Dhau I^6^, Maimela E^1,7^

^1^ DIMAMO Population Health Research Centre, University of Limpopo, Sovenga 0727, Polokwane 0700, South Africa;

^2^Department of Nursing Science, University of Limpopo, Sovenga 0727, Polokwane 0700, South Africa;

^3^Department of Computer Science, University of Limpopo, Sovenga 0727, Polokwane 0700, South Africa;

^4^Research Administration and Development, University of Limpopo, Sovenga 0727, Polokwane 0700, South Africa;

^5^Department of Pharmacy, University of Limpopo, Sovenga, 0727, Polokwane, 0700, South Africa;

^6^Department of Geography and Environmental Studies, University of Limpopo, Private Bag X1106, Sovenga 0727, Polokwane, South Africa;

^7^Department of Public Health, University of Limpopo, Sovenga 0727, Polokwane 0700, South Africa

*** Correspondence:**Ntimana Cairo Bruce

[**cairo.ntimane@ul.ac.za**](mailto:cairo.ntimane@ul.ac.za)

**Seakamela Kagiso Peace**

[**kagiso.seakamela@ul.ac.za**](mailto:kagiso.seakamela@ul.ac.za)

**Appendix 1:** **Depression score questions developed from the literature.**

|  |  | **Answer options** | | | |
| --- | --- | --- | --- | --- | --- |
| **Value** | **Question** | **Option 1 (score)** | **Option 2 (score)** | **Option 3 (score)** | **Option 4 (score)** |
| C1 | I’ve been able to laugh and see the funny side of things | As much as I always could (0) | Not quite so much now (1) | Definitely not so much now (2) | Not at all (3) |
| C2 | I have looked forward with enjoyment to things | As much as I ever did (0) | Rather less than I used to (1) | Definitely less than I used to (2) | Hardly at all (3) |
| C3 | I have blamed myself unnecessarily when things went wrong | Yes, most of the time (3) | Yes, some of the time (2) | Not very often (1) | No never (0) |
| C4 | I have been anxious or worried for no good reason | No, not at all (0) | Hardly ever (1) | yes sometimes (2) | Yes, very often (3) |
| C5 | I have felt scared or panicky for no very good reason | quite a lot (3) | Yes, sometimes (2) | No, not much (1) | No , not at all (0) |
| C6 | Things have been getting too much for me | Yes, most of the time I haven’t been able to cope at all (3) | Yes, sometimes I haven’t been coping as well as usual (2) | No, most of the time I have coped quite well (1) | No, I have been coping as well as ever (0) |
| C7 | I have been so unhappy that I have difficulty sleeping | Yes, most of the time (3) | Yes, sometimes (2) | Not very often (1) | No, not at all (0) |
| C8 | I have felt sad or miserable | Yes, most of the time (3) | Yes, quite often (2) | Not very often (1) | No, not at all (0) |
| C9 | I have been so unhappy that I have been crying | Yes, most of the time (3) | Yes, quite often (2) | Only occasionally (1) | No, never (0) |
| C10 | The thought of harming myself has occurred to me | Yes, quite often (3) | Sometimes (2) | Hardly ever (1) | Never (0) |
